# Thoracic Segmental Anesthesia for Major Laparoscopic Abdominal Surgery in a Heart Transplant Recipient: A Case Report

**DOI:** 10.7759/cureus.74309

**Published:** 2024-11-23

**Authors:** Benedetta Basta, Davide Vailati, Luigi Mori, Carmelo Magistro, Giovanni Marino

**Affiliations:** 1 Anesthesia and Intensive Care, Melegnano Hospital - ASST Melegnano e Martesana, Milan, ITA; 2 General Surgery, Melegnano Hospital - ASST Melegnano e Martesana, Milan, ITA

**Keywords:** awake neuraxial surgery, colorectal laparoscopic surgery, heart transplant patient, intrathecal dexmedetomidine, neuraxial anesthesia, segmental thoracic spinal anesthesia

## Abstract

We describe the case of a 72-year-old male suffering from Marfan syndrome, who, because of cardiac abnormalities correlated to the syndrome, received an orthotopic heart transplant four years ago. In 2024, he was diagnosed with right colon cancer. The decision to operate was difficult because of the elevated perioperative risk. Focused on the possible cardiologic and respiratory complications, we decided to proceed with neuraxial anesthesia during spontaneous breathing. This emergent anesthesiological strategy for open and laparoscopic abdominal surgery has the potential to abolish many risks related to general anesthesia. Right laparoscopic hemicolectomy was successfully performed, and no medical or surgical complications occurred in the postoperative period.

## Introduction

In the surgical population, comorbidity, aging, and frailty are increasingly represented [1]. The decision to operate is sometimes challenging due to difficulty balancing risks versus benefits, especially in the case of cancer. This is particularly relevant for a post‑cardiac transplant recipient, such as the patient described in this paper, necessitating abdominal surgery. In this patient population, the main concerns involve altered autonomic physiology and response to medications because of denervation of the donor's heart [2], as well as immunodepressive state because of immunomodulatory therapy [2], as we will explain.

Many efforts have been undertaken in recent years to minimize the anesthesiological and surgical impact on the physiological status and to reduce perioperative complications. In this context, a possible anesthesiological strategy for abdominal surgery is the so-called 'awake neuraxial surgery' that combines neuraxial anesthesia and mild sedation, keeping the patient in spontaneous breathing. This emergent approach, which is old but has been renewed especially during the COVID-19 pandemic period [3], aims to counteract all the potential impairments produced by general anesthesia [4].

## Case presentation

We describe the case of a 72-year-old male suffering from Marfan syndrome, a hereditary disorder of the connective tissue that results in abnormalities of the eyes, bones, heart, blood vessels, lungs, and central nervous system [5]. The patient had developed severe dilatative cardiomyopathy, and in 2020, received a heart transplant. Cardiologic assessment confirmed good cardiac compensation and contractile activity. The patient also reported recurrent episodes of pneumothorax because of his syndrome. Some episodes of ischemic strokes occurred because of thrombophilia due to hyperhomocysteinemia, even though without serious sequelae expected of tremor. Additionally, he had developed chronic renal failure. In February 2024, he was diagnosed with colon cancer, and a right hemicolectomy was indicated. The decision to operate was challenging.

He was certainly a so-called 'high-risk patient'. All worries arose about altered autonomic physiology and response to medications because of the denervation of the donor's heart. The donor's heart had a higher resting heart rate (90-110 beats/min) that could not change as an adaptative response because of the absence of parasympatic innervation, resulting in being extremely sensitive to changes in venous return [2]. Furthermore, a certain alarm was detected because of ongoing immunosuppressive therapy consisting of a calcineurin inhibitor (cyclosporine) together with an antimetabolite agent (mycophenolate). After discussing with the transplant center where the patient was following up, it was concluded that immunosuppressive therapy could not be stopped, even in the case of major abdominal surgery. In consideration of immunosuppressive state, strict aseptic techniques, minimum use of indwelling catheters, and earliest removal of invasive lines were pursued and attentive clinical and laboratory infection monitoring was observed during and after hospital stay.

Less troubling was the management of anti-aggregant therapy that the patient was taking because of past neurological ischemic events due to hyperhomocysteinemia and thrombophilia state; acetylsalicylic acid 100 mg oral therapy was maintained and thromboprophylaxis with low molecular weight heparin was applied according to local protocol.

However, a high risk remained for thrombotic events. A high risk also remained for pneumothorax in case of mechanical ventilation, concerning lung abnormalities of Marfan syndrome, and metabolic complications because of chronic renal failure.

After a college multidisciplinary meeting aiming to explore strategies to minimize perioperative risk, we decided to approach the patient with thoracic neuraxial anesthesia. The patient was prepared according to the local enhanced recovery after surgery (ERAS) protocol [6] 14 days before scheduled surgery.

Comprehensive anesthesia monitoring included electrocardiogram, pulse oximetry, invasive arterial pressure, semi-invasive cardiac monitoring (ProAQT/PulsioFlex), bispectral index (BIS), capnography, and body temperature.

Given the need to obtain anesthetic coverage for right colon resection in a laparoscopic approach, we performed thoracic spinal anesthesia at T9-T10 level, with levobupivacaine 10 mg and dexmedetomidine 5 mcg mixed in the same syringe in a single shot (total volume 4 ml) (see Table 1 in Appendices). Dexmedetomidine was used as an adjuvant to increase the block's duration and extension [7]. Written informed consent was obtained for dexmedetomidine off-label intrathecal use. The sensory anesthetic level was achieved at the T2 level and was proven by pin-prick test. Before thoracic spinal puncture, an epidural catheter was placed at T7-T8 level and used for postoperative analgesia and during surgery as needed. In this case, lidocaine 1% boluses of 5 ml (total 10 ml) were administered at the beginning of surgery. Intravenous sedation was obtained by dexmedetomidine IV infusion along with ketamine boluses during surgically sensitive moments (see Table 2 in Appendices). The sedation goal was a Richmond Agitation-Sedation Scale (RASS) score of 0/-2. Hypotension occurred soon after intrathecal injection, which was promptly treated with norepinephrine infusion. No impact on cardiac output was detected. Norepinephrine IV infusion was necessary throughout the surgery up to a maximum of 0.2 mcg/kg/min and was stopped gradually four hours after the end of the intervention. Goal-directed fluid therapy was applied according to stroke volume variation. Spontaneous breathing was maintained throughout the procedure, with an oxygen mask of 2-3 L/min. Blood gas arterial samples were collected at baseline and at every hour in order to monitor the partial pressure of arterial oxygen/fraction of inspired oxygen (P/F) ratio that was always >300, partial pressure of carbon dioxide (pCO_2_) level, and acid-basis status; no alterations were detected. The surgery lasted two hours and was performed by laparoscopy with maximal abdominal pressure of 12 mmHg; no surgical troubles occurred and surgeons were able to operate with a high degree of satisfaction. Immediately after the dexmedetomidine IV infusion stopped, the patient was perfectly arousable. Cautionary hospitalization in an intensive care setting was planned. The epidural catheter provided postoperative analgesia (starting at the end of surgery) through the administration of ropivacaine 0.2% at a dosage between 4-6 ml/hour. No medical or surgical complications occurred during the postoperative period. The patient was able to ambulate on the first day postoperatively and was discharged after four days. Written informed patient consent was obtained for the publication of this case report.

## Discussion

Heart-transplanted patients represent a rare population with clinically complex features [2]; these patients may require anesthesia for elective or emergency surgery and proper strategy is not so easy to define. It is mandatory to consider that a transplanted heart that was denervated has clinical implications: resting heart rate (90-110 beats/min) is higher, adaptative tachycardia in case of hypotension is not present, and anticholinergic drugs such as atropine have no effects [2]. On the other side, intrinsic cardiac mechanisms are preserved, resulting in the heart being extremely sensitive to changes in loading conditions [2]. These patients are at higher risk of developing cardiac arrhythmia, ischemic events, and cardiac arrest in the perioperative period [2]. Other concerns are about immunosuppressant therapy that exposes to the risk of infections [2]. All anesthetic techniques have been used successfully in these patients. No technique has been demonstrated to be better as long as care is given to maintain adequate preload [8]. General anesthesia is commonly preferred due to worries of impaired response to hypotension after spinal or epidural anesthesia, even in the absence of documented data [8].

Unlike common practice, we preferred to avoid general anesthesia and approached the case described by thoracic segmental anesthesia (TSA). This is an emergent anesthesiological strategy for open and laparoscopic abdominal surgery [9], used also in breast and thoracic surgery, often proposed in high-risk patients or alternatively in low-resource settings [10,11].

TSA, defined as an intrathecal injection at the thoracic level above L1-L2 by targeting particular spinal segments according to surgical requirements, is able, as most locoregional techniques, to avoid some drawbacks of general anesthesia [4]. Many potential benefits of spinal anesthesia compared to general anesthesia include: avoidance of airway manipulation and positive pressure ventilation, stable hemodynamics, less blood loss, easy homeostasis of carbon dioxide in spontaneously breathing awake patients, opioid-sparing, better intraoperative and postoperative pain control, earlier recovery of gastrointestinal function, less postoperative nausea and vomiting, lower incidence of deep vein thrombosis, lower incidence of surgical site infections, shorter stay in post-anesthesia care, and earlier discharge from hospital [4,12].

With respect to lumbar anesthesia, TSA is more predictable in anesthetic level achieved when the thoracic block is required and could employ reduced total intrathecal dose [4], leading to more stable hemodynamics [13].

We used levobupivacaine for thoracic spinal anesthesia due to its less cardiotoxicity and reduced central nervous system effects in comparison with other local anesthetics [14]. The final concentration obtained with saline was 0.25%, sufficient to obtain an anesthetic effect and long duration because of the addition of dexmedetomidine [15]. A mild hypobaric mixture was obtained, and an intrathecal injection of 4 ml volume was considered sufficient to interest the metamers involved in laparoscopic abdominal surgery, according to previous studies [16].

Good surgeon satisfaction and patient comfort during TSA were recorded. Thus, all considerations make TSA fit for high-risk patients [17] as a means to reduce systemic complications related to general anesthesia, with special regard, concerning this case, to respiratory, hemodynamic, and thrombotic ones.

Even though an attractive strategy, it is important to emphasize the significance of careful patient selection and trained anesthesiologist expertise for successful outcomes [18]. A context of anesthesia-monitored care and defined protocol must be ensured concerning high-risk patient profiles and anesthetic technique peculiarity [15].

## Conclusions

Heart transplant recipients represent a particularly high-risk population in which elective surgery could be necessary, making anesthesiological strategy crucially important. TSA, as an alternative to general anesthesia, has been performed for abdominal surgery in different populations but never in the context of a heart transplant. This is the first time that this technique has been successfully applied in such a setting. Potential advantages include avoiding airway manipulation, reducing risks associated with mechanical ventilation, and achieving more stable hemodynamics. This case report reveals a successful outcome and high satisfaction from both the surgeon and the patient. Even though careful perioperative management is required for heart transplant recipients, TSA, in the context of advanced monitored anesthesia care, could represent an effective and safe option.

## Appendices

**Table 1:** IV sedation regimen

Drug	Route of Administration	Dose
Dexmedetomidine	IV continous infusion	0.2-1 mcg/kg/h
Ketamine	IV bolus (10-20 mg)	100 mg

**Table 2:** Intrathecal treatment

Drug	Dose	Volume
Levobupivacaine	10 mg	2 ml
Dexmedetomidine	5 mcg	1 ml
Sodium chloride solution 0.9%	9 mg	1 ml
